# Serologic Evidence of Human Granulocytic Ehrlichiosis, Greece

**DOI:** 10.3201/eid0806.010500

**Published:** 2002-06

**Authors:** Stella Alexiou Daniel, Katerina Manika, Malamatenia Arvanitidou, Eudoxia Diza, Nikolaos Symeonidis, Antonis Antoniadis

**Affiliations:** Aristotelian University of Thessaloniki, Thessaloniki, Greece

To the Editor: Human granulocytic ehrlichiosis (HGE), a tickborne infectious disease, was first described in 1994 (*1*). Several cases have been reported in the United States; reports of acute cases in Europe have been rare, although European serosurveys of the prevalence of antibodies to the HGE agent have been conducted (*2*–*4*). No similar serosurvey has been conducted in Greece, although *Ixodes ricinus*, thought to be the principal tick vector in Europe (*5*), is present in northern Greece (*6*). Lyme disease, which is transmitted by the same tick, has never been reported, and the seroprevalence of Lyme borreliosis in Greece is very low (*7*).

We examined sera of 300 persons (100 men and 200 women) ages 15–78 years (mean age ± standard deviation 52.7±18.0 years), which were collected at six county hospitals in northern Greece and sent to our laboratory from April to October 2000. The participants were mostly farmers, all of whom lived in rural areas of northern Greece. All participants were healthy and had been hospitalized for routine blood tests. Each patient completed a questionnaire about medical history. The selected patients had no known history of rickettsiosis and reported no febrile or influenza-like illness during the past 6 months. Each participant provided oral consent for the serum to be used for detecting antibodies against several infectious agents related to zoonoses. The following information was recorded for each participant: age, sex, occupation, and area of residence.

Serum samples were tested by indirect immunofluorescence (IFA) with commercially available antigen (Focus Technologies, Cypress, California), which uses HGE-1–infected HL60 cells. Titers >64 were considered positive. All sera were also tested for *Rickettsia conorii*, *R. typhi*, *Coxiella burneti,* and *Ehrlichia chaffeensis* by IFA and for *Borrelia burgdorferi* by enzyme-linked immunosorbent assay and Western blot. Sera that reacted positively to more than one of these agents were excluded. Biostatistical analysis was performed by using the statistical package SPSS for Windows 10.0.1 (Standard version, SPSS Inc., Chicago, IL).

The overall prevalence of antibodies to the HGE agent was 7.3% (8.0% for men and 7.0% for women). No statistically significant differences were observed in the prevalence of antibodies in the six prefecture hospitals. Participants had no statistically significant differences in sex or age. Antibody titers to HGE were low (of 22 positive sera, 12 had titers >64 and 10 had titers >128).

Several serosurveys of the prevalence of antibodies to the HGE agent have been conducted across Europe in both healthy persons and patients with suspected or confirmed Lyme borreliosis (*2*,*3*,*8*). Since cases of *B. burgdorferi* infection are rare or nonexistent in Greece and the seroprevalence of Lyme borreliosis is very low, we selected as participants 300 healthy farmers who lived in rural areas. These persons compose a group at high risk for exposure to tick bites and therefore to *I. ricinus*. Our prevalence is higher than those observed in Bulgaria (2.9%) and Germany (1.9%) (*2*,*3*). This finding could be attributed to the fact that the prevalence in these countries was based on blood donors, unlike our survey. However, our prevalence is substantially lower than that in Slovenia, where 15.4% of the examined population had detectable antibodies to the HGE agent and several cases of HE have been confirmed (*4*). Our observation that no significant differences occurred in the prevalence of antibodies to the HGE agent in the six prefectures studied could be explained by the fact that these districts are small, with little variation in environmental and climatic conditions. Even though the antibody titers to the HGE agent were low in our survey, they suggest infection at an undetermined time (*9*). Seven of our sera were antibody positive to both the HGE agent and at least one other rickettsial agent or *B. burgdorferi*. This fact, which has been observed elsewhere (*9*), may result from coinfection or crossreaction. These sera were excluded. Our data suggest the possibility that HGE cases exist in Greece. Since such cases have been not been reported to date, they are likely underdiagnosed. Further research is needed to clarify the presence of the HGE agent in Greece.
